# An Interspecific Assessment of Bergmann’s Rule in Tenebrionid Beetles (Coleoptera, Tenebrionidae) along an Elevation Gradient

**DOI:** 10.3390/insects15090673

**Published:** 2024-09-05

**Authors:** Simone Fattorini

**Affiliations:** Department of Life, Health and Environmental Sciences, University of L’Aquila, Via Vetoio, 67100 L’Aquila, Italy; simone.fattorini@univaq.it

**Keywords:** Bergmann’s rule, beetles, body size, body volume, darkling beetles, elevation, thermoregulation

## Abstract

**Simple Summary:**

Warm-blooded animals (like mammals and birds) living in colder climates (higher latitudes or elevations) tend to have larger bodies compared to their relatives in warmer areas. This pattern has been interpreted in terms of heat budget: since the surface area-to-volume ratio decreases with increasing size, larger animals need to produce relatively less heat to maintain stable body temperatures (Bergmann’s rule). This mechanism cannot operate in cold-blooded animals, like most insects, as they do not produce heat. In these animals, a reverse pattern is frequently observed, as an increased surface area-to-volume ratio may allow for more rapid heating and cooling. However, selection for more stable internal temperatures might lead to smaller surface area-to-volume ratios also in cold-blooded animals, leading to a negative correlation between body size and temperature. An analysis conducted on tenebrionids along an elevational gradient in Central Italy revealed that, on average, their surface area-to-volume ratios decline with increasing elevation, thus indicating the importance of heat conservation. However, while in species living under bark or in rotten wood, body size (mass and volume) tends to increase with elevation, in ground-dwelling species, it declines up to about 1000 m and then increases. This suggests that a reduction in resource availability with increasing elevation limits body size in ground-dwelling species up to a certain elevation but not in those living under bark, which benefit from more microclimatically stable conditions and constant resources and need energy for overwintering.

**Abstract:**

In endotherms, body size tends to increase with elevation and latitude (i.e., with decreasing temperatures) (Bergmann’s rule). These patterns are explained in terms of heat balance since larger animals need to produce less heat relative to their size to maintain stable body temperatures. In ectotherms like most insects, where this mechanism cannot operate, a reverse pattern is frequently observed, as a higher surface area-to-volume ratio in colder climates may allow for more rapid heating and cooling. However, patterns of increasing body size with decreasing temperatures can also be observed in ectotherms if selection for more stable internal temperatures leads to smaller surface area-to-volume ratios. Data on tenebrionids from Latium (Central Italy) were used to model elevational variations in average values of body size (total length, mass and volume) and surface area-to-volume ratio. Analyses were performed by considering the whole fauna and two ecological groups separately: ground-dwelling species (geophilous) and arboreal (xylophilous) species. The surface area-to-volume ratios declined with increasing elevation in all cases, indicating that the need for heat conservation is more important than rapid heating and cooling. However, in xylophilous species (which typically live under bark), body size increased with increasing elevation, and in geophilous species, an opposite pattern was observed up to about 1000 m, followed by an increasing pattern. This suggests that a reduction in resource availability with elevation limits body size in geophilous species up to a certain elevation but not in xylophilopus species, which benefit from more climatically stable conditions and constant resources and need energy for overwintering.

## 1. Introduction

In its broadest definition, Bergmann’s rule [1] describes an ecogeographical pattern in which animals living in colder climates tend to have larger bodies than their relatives living in warmer areas [2,3]. Bergmann’s rule is one of the most investigated and debated ecogeographical rules [2,4,5,6,7,8,9,10,11,12,13]. The rule was initially conceived for endothermic animals (and explicitly tested in birds) [1] because of the advantage that a larger size may have in terms of heat budget in cold climates. The mechanism hypothesized by Bergmann can be summarized as follows: (1) since in endothermic animals the surface area determines the rate of heat dissipation, while the volume determines heat production, the ratio between surface area and volume determines their thermoregulatory ability; (2) with increasing body size, the surface area of an animal increases less than its volume; thus, the surface area-to-volume ratio decreases; (3) as a result, larger animals need to produce less heat in relation to their size to raise their temperature above the ambient temperature, which (4) will favor larger bodies in colder climates [1].

Although some authors suggest that the expression “Bergmann’s rule” should be used to indicate this mechanism, and not the ecogeographical pattern (which might have other explanations) [10,14], in the present paper, it will be used, in accordance with common practice, to indicate the pattern of increasing size with increasing latitude or elevation, independently from the possibly underlying mechanisms. The expression “Bergmann’s hypothesis” will be used to indicate the thermoregulatory explanation.

It has been long debated if (1) Bergmann’s rule and Bergmann’s hypothesis apply to intraspecific or interspecific comparisons (or both) [2,6,15,16,17], (2) they should be applied only to latitudinal gradients or can be valid also along elevational gradients, as temperature varies regularly along both gradients [17], and (3) ectothermic animals might follow Bermann’s rule [17,18,19,20,21,22,23,24,25].

Studies on Bergmann’s rule in ectotherms produced largely idiosyncratic results, with an opposite trend (i.e., decreasing body size in colder climates) being frequently recovered (a pattern sometimes indicated as “converse Bergmann”) [17,20,26,27,28,29,30]. In ectothermic animals, smaller body sizes may be favored in colder climates as an increased surface area-to-volume ratio allows for more rapid heating and cooling, which may be a thermal explanation for the converse Bergman rule [19,31]. On the other hand, selection for more stable internal temperatures should lead to smaller surface area-to-volume ratios [19], producing patterns consistent with Bergmann’s rule also in ectotherms. Thus, the basic idea of Bergmann’s hypothesis, that larger bodies are favored in cold climates as the lower surface area-to-volume ratio reduces heat dissipation can also be applicable to ectotherms, when the benefits of increased conservation of heat due to a larger body size overpay for the decreased capacity to heating up due to a larger body mass.

Research on insects (most of which are ectotherms) found patterns conforming to Bergmann and converse Bergmann patterns with similar frequencies, with results varying according to the taxon, species ecology, and study design (latitudinal vs. elevational data, inter- vs. intraspecific analyses, type of body size measures, such as total body length, volume or biomass, measures of certain organs, etc.) [17,20,27,29,32]. In particular, the presence of converse Bergmann patterns in insects has been explained with reference to the heat-dependence of growth and metabolic rates in ectotherms [33,34] and the earlier diapause and shorter growing seasons of insects living in cold climates [35].

Most research investigated how insect body size varies along latitudinal and elevational gradients using linear measures (e.g., length of total body or of specific body parts, such as leg segments), whereas only a minority of studies used body volume and mass [17]. To the best of my knowledge, apart from a study exploring the intraspecific variation of the surface area-to-volume ratio with latitude in some Phasmatodea [36], no research investigated how this ratio varies along latitudinal or elevational gradients in insects. This is a particularly significant gap, as the use of the surface area-to-volume ratio allows for the most direct testing of Bergmann’s rule [36]. The aim of the present paper is therefore to investigate how average body size and surface area-to-volume ratio vary in a group of beetles along an elevational gradient at a regional level.

Although variation in temperature is an obvious feature of elevational gradients, resource availability also decreases with increasing elevation [37,38], which may influence body size, as larger bodies need more energy (and hence availability of more resources) for growth [32,39]. Based on these considerations, four alternative hypotheses can be formulated about the relative importance that different thermoregulation processes and resource availability may have in generating patterns of average body size of insect species assemblages along elevational gradients.

**H1.**  *The need for heat conservation [19] is more important than rapid heating and cooling [19,31], and resource availability limits body size [32,39]. This hypothesis predicts that both the surface area-to-volume ratio and body size should decrease with elevation*.

**H2.**  *The need for rapid heating and cooling [19,31] is more important than heat conservation [19], and resource availability limits body size [32,39]. This hypothesis predicts that the surface area-to-volume ratio should increase with elevation while body size should decrease.*

**H3.**  *The need for heat conservation [19] is more important than rapid heating and cooling [19,31], and resources do not limit body size (e.g., if resources are not limited by declining temperatures). This hypothesis predicts that the surface area-to-volume ratio should decrease with increasing elevation, while body size might show either no pattern or a positive trend if having a larger size may be beneficial for any reason.*

**H4.**  *The need for rapid heating and cooling [19,31] is more important than heat conservation [19], and resources do not limit body size. This hypothesis predicts that the surface area-to-volume ratio should increase with elevation, whereas body size might show no pattern or a positive trend.*

In the present paper, I tested these hypotheses for tenebrionid beetles (Coleoptera Tenebrionidae) along a regional elevational gradient of 2400 m in central Italy.

## 2. Materials and Methods

I considered the elevational distribution of tenebrionid beetles from the Latium Region, central Italy. This region extends for about 17,200 km^2^ in the central part of the Italian peninsula. The region, which fronts the Tyrrhenian Sea, is largely dominated by hilly and mountain landscapes (central Apennines), rising to 2458 m at Monte Gorzano. Temperatures decline considerably from lowland areas to mountain tops, whereas low-elevation areas are characterized by a Mediterranean climate, with annual temperatures even around 17 °C, scarce annual rainfall (<1000 mm) and a clear period of summer aridity. The highest peaks show subalpine climates, with average annual temperatures < 10 °C, abundant rainfall (ca 1500 mm) and a lack of a period of summer aridity [40,41]. Vegetation also varies distinctly with elevation, from dune, Mediterranean shrubland and maquis vegetation along the coast to pseudo-alpine grasslands beyond the tree line [42]. Thanks to more than one century of intensive entomological research, the tenebrionid fauna of this area is relatively well known [42,43,44,45], thus representing an ideal system to investigate body size variation along an elevation gradient at a regional level.

The whole elevational gradient was divided into 24 belts of 100 m (0–100, 101–200, 201–300 m, etc.), and presence/absence in each belt was established for 85 native species/subspecies of tenebrionid beetles [42,46]. I considered both species and subspecies because the dividing line between these two categories is largely arbitrary in tenebrionid beetles, as shown by many former subspecies recently elevated to species level [47,48,49,50,51,52]. For simplicity, the term species will be used here to indicate both ranks.

The genus *Lagria* (traditionally assigned to the former family Lagriidae, now a subfamily of Tenebrionidae) and the subfamily Alleculinae (formerly considered a separate family) were not considered because their lifestyle (they are typically associated with flowers and leaves) is completely different from that of all other tenebrionids, and their distribution in the study area is still poorly known [42,44]. I also omitted synanthropic species associated with human food (and which became cosmopolitan or subcosmopolitan) as well as alien species introduced into Italy [42,44]. 

Nomenclature followed Iwan and Löbl [53]. As the separation of *Leptoderis italicus* Ferrer 2015 from *Leptoderis collaris* (Linnaeus, 1767) [54] and *Pachychila italica* Ferrer, 2018 from *Pachychila frioli* Solier, 1835 [55] is equivocal, these taxa are listed under *L. collaris* and *P. frioli*, respectively.

Species distribution across the elevational gradient was taken from Fattorini [44] and Fattorini et al. [46], with the addition of *Eledonoprius armatus* (Panzer, 1799) [56]. Species were divided into two main groups according to their lifestyle: geophilous species (ground-dwelling species, usually found under stones or in the sand, mainly under debris) and xylophilous species (species mainly associated with trees, being especially found under bark and in rotten wood), following Fattorini [42]. For a few species that can occupy both habitats, the preferred habitat was considered [42].

The study followed an assemblage approach analysis [57], in which values of the body size (length, mass and volume) and the surface area-to-volume ratio of the species occurring at a certain elevation were averaged and regressed against elevation.

Species body lengths (total length, tl, defined as the distance between labrum and end of the elytra, in mm) were taken from Fattorini et al. [58].

Species biomass was estimated using the relationship between body length and biomass reported by Fattorini [59] for fresh tenebrionids:

Bf = 0.0197tl^3.408^, (1)

where Bf is the fresh biomass in mg, and tl is the total body length in mm.

As an alternative measure of body mass, dry body mass was estimated using the equation for tenebrionids reported by Hódar [60]:

Bd = 0.051tl^2.669^, (2)

where Bd is the dry biomass in mg, and tl is the total body length in mm as above.

Following Kuschka [61], species body volumes were estimated by approximating the tenebrionid body to an ellipsoid using the formula:

V = π/6 × tl × mw × mh,(3)

where tl was the total body length defined as above, mw was the cross-body distance (maximum elytral width, in mm), and mh was the height (maximum thickness of the beetle body, in mm).

Measures of maximum width and maximum height were obtained by measuring museum specimens and then recalculated proportionally to the average length values given in Fattorini et al. [58]. Measurements were conducted using a caliper, macrophotographs or microphotographs, depending on the size of the species.

These measures were also used to calculate the body surface, using the following formula, which represents a reasonable approximation of an ellipsoid surface [62]:

S = 4π[(a^p^ × b^p^ + a^p^ × c^p^ + b^p^ × c^p^)/3]^1/p^, (4)

where p = 1.6075, and a, b and c correspond to tl/2, mw/2 and mh/2, respectively.

Average values of species body length, body mass (fresh and dry), volume and surface area-to-volume ratio were calculated for each elevational band and regressed against elevation (middle point of the elevation band). OLS regressions were used. Calculations were conducted in R 4.1.3 software [63]. Graphs were constructed with the R package ggplot2 [64]. Species distribution across the elevational gradient, lifestyle and body measures are reported in Table S1. Average values of species body length, body mass (fresh and dry), volume and surface area-to-volume ratio are reported in Table S2.

## 3. Results

**Figure 1:**
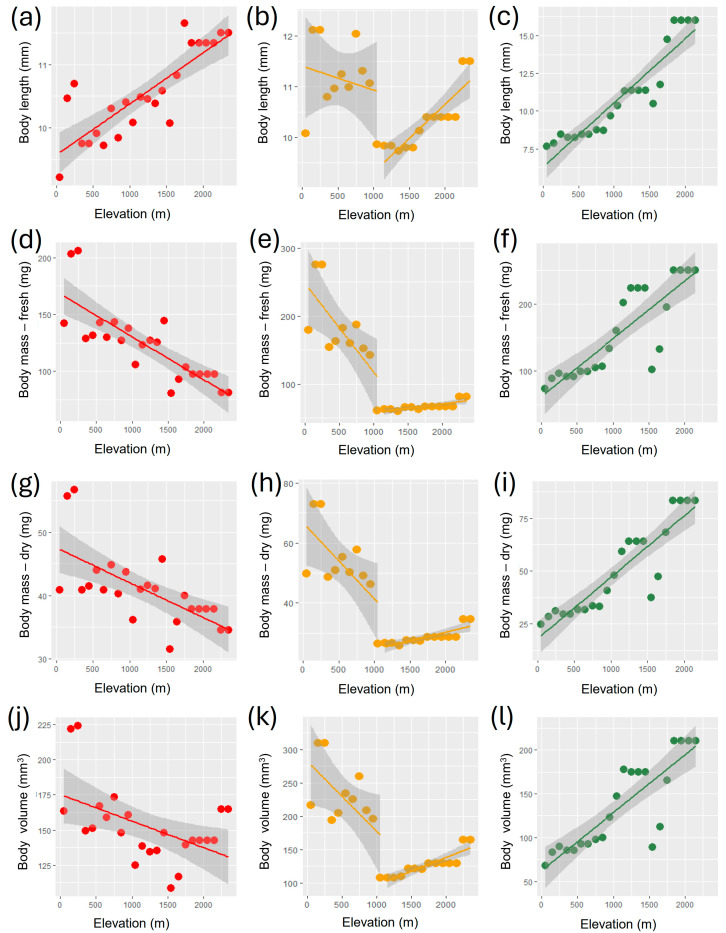
Relationships (regression lines) of average values of body length, body mass and body volume with elevation for tenebrionid beetles in Latium (Central Italy): (**a**) relationship between average body length and elevation for all species together; (**b**) relationship between average body length and elevation for geophilous species; (**c**) relationship between average body length and elevation for xylophilous species; (**d**) relationship between average body mass (fresh) and elevation for all species together; (**e**) relationship between average body mass (fresh) and elevation for geophilous species; (**f**) relationship between average body mass (fresh) and elevation for xylophilous species; (**g**) relationship between average body mass (dry) and elevation for all species together; (**h**) relationship between average body mass (dry) and elevation for geophilous species; (**i**) relationship between average body mass (dry) and elevation for xylophilous species; (**j**) relationship between average body volume and elevation for all species together; (**k**) relationship between average body volume and elevation for geophilous species; (**l**) relationship between average body volume and elevation for xylophilous species. For geophilous species, regression lines are shown for the two segments of the elevational gradient analyzed separately (below 1100 m and above 1100 m); overall regression lines without this distinction are shown in Figure S1.

When all species were considered together, average body length increased with elevation (Table 1, Figure 1a). However, when analyzed separately, geophilous and xylophilous species showed different patterns. The average body length for geophilous species did not show a significant monotonic relationship with elevation but presented two groups of data with different patterns, with a significantly positive relationship above 1100 m (Table 1, Figure 1b and Figure S1a). By contrast, a positive relationship was observed for xylophilous species (Table 1, Figure 1c).

The average body mass (fresh) for all species together decreased with increasing elevation (Table 1, Figure 1d). However, also in this case, when analyzed separately, geophilous and xylophilous species showed different patterns. Geophilous species showed a global negative relationship (Table 1, Figure S1b), but the pattern was, in fact, composite, with a negative relationship below 1100 m and a positive relationship above this elevation (Table 1, Figure 1e). By contrast, body mass for xylophilous species increased with elevation (Table 1, Figure 1f). The use of dry body mass produced analogous results (Table 1, Figure 1g–i and Figure S1c).

The average body volume for all species together (Table 1, Figure 1j) decreased with increasing elevation. When analyzed separately, geophilous and xylophilous species again showed different patterns. Geophilous species showed a global negative relationship (Table 1, Figure S1d), but the pattern was, in fact, composite, with a negative relationship below 1100 m and a positive relationship above this elevation (Table 1, Figure 1k). Average body volume for xylophilous species increased with elevation (Table 1, Figure 1l).

**Figure 2:**
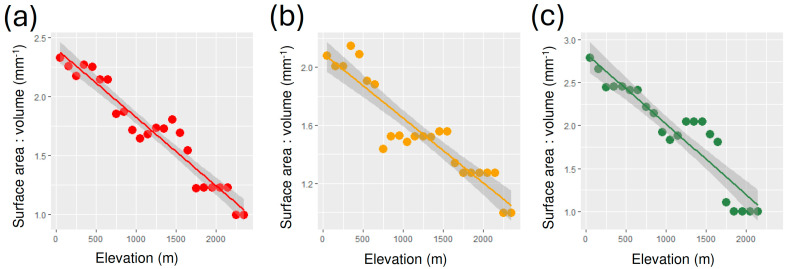
Relationships between surface area-to-volume ratio and elevation for tenebrionid beetles in Latium (Central Italy): (**a**) relationship between average surface area-to-volume ratio and elevation for all species together; (**b**) relationship between average surface area-to-volume ratio for geophilous species; (**c**) relationship between average surface area-to-volume ratio and elevation for xylophilous species.

Average surface area-to-volume ratios decreased with elevation for total species (Table 2, Figure 2a), geophilous species (Table 2, Figure 2b) and xylophilous species (Table 2, Figure 2c).

The negative relationships of surface area-to-volume ratio and body size (mass or volume) with increasing elevation support the hypothesis (H1) that heat conservation is more important than rapid heating and cooling and that resources limit body size for the geophilous species (up to about 1000 m) and for all tenebrionid species together. The reduction in surface area-to-volume ratio associated with a positive relationship between average body size and elevation supports the hypothesis (H3) that heat conservation is more important than rapid heating and cooling and resource availability does not limit body size in xylophilous species and in geophilous species above 1100 m. No support was found for the other two hypotheses (i.e., H2: rapid heating and cooling is more important than heat conservation, resource availability limits body size; H4: rapid heating and cooling is more important than heat conservation, resource availability does not limit body size).

## 4. Discussion

The study of variation in body size in tenebrionid beetles along an elevational gradient in central Italy produced partially contrasting results, depending on the type of measure used (body length, body mass or body volume) and the species assemblage considered (all species together, geophilous species, or xylophilous species). When all species were considered together, a positive relationship with elevation (Bergmann’s rule) was found for body length, but the use of body mass (fresh or dry) or body volume led to negative relationships (converse Bergmann). For geophilous species, a negative (converse Bergmann) pattern was detected for all types of body size measure (although relationship with body length was not significant, and for body length, mass and volume the negative relation turned into a positive one above 1100 m), while a positive (Bergmann) pattern was found for all types of measures of body size for xylophilous species. Surface area-to-volume ratios showed negative relationships with elevation for both total species richness and the two ecological categories (geophilous species and xylophilous species) analyzed separately.

Previous research in tenebrionid beetles showed a latitudinal decrease in average body length across European countries, with climate acting as an important filtering factor that determines the prevalence of larger species in southern areas [58,65]. This means that larger species occurring at lower latitudes (warmer areas) tend to be filtered out with increasing latitudes (i.e., with decreasing temperatures). Although a filtering process also operates on the elevational distribution of tenebrionid beetles in Latium, with species of higher elevations being mostly subsets of those living at lower elevations [44], a positive relationship was found between average body length of total tenebrionid species and elevation (thus, an increase in average body length with decreasing temperature).

This positive relationship found for the body length is consistent with Bergmann’s rule, but it contrasts with the results obtained using body mass and body volume. These contrasting results may be explained on a statistical basis due to the different impacts that large species have when different types of measures are used. Larger species, which are a minority in the studied fauna [66], have a negligible influence on the calculation of average values when linear measures are used. However, their impact is emphasized in the calculation of weights and volumes because quadratic and cubic measures are used here. As most of the larger tenebrionids are geophilous species associated with lowland areas (e.g., *Pimelia cajetana*, *Blaps* spp., *Akis* spp., etc.), this can explain the particular shape of the relationship found for this ecological group: a rapid decline in body mass and volume up to 1000 m, with only much smaller species above this elevation. This is consistent with the hypothesis that large body sizes are favored by high temperature and low precipitation [65] and negatively affected by resource-limited conditions occurring at high elevations, where colder conditions lead to shorter growing seasons and reduced overall productivity [37]. Under such conditions, small-sized species could be favored, as they can survive with less food intake [39,67,68]. Overall, the declining average body mass and volume in geophilous tenebrionids parallels the latitudinal decrease observed in the average body length of tenebrionids in Europe, where high temperatures and aridity were identified as important factors favoring large-sized beetles at low latitudes [65]. However, above 1100 m (a turning point in tenebrionid species richness and composition in the study area [44]), geophilous species showed an increasing average body mass and volume with increasing elevation, thus indicating that—above a certain elevation, and hence below a body of certain size—resources are no more a limiting factor, and having a larger body size may be advantageous.

Differently from geophilous species, the average body size of xylophilous species increased with elevation for all measures. Beetles that live under bark, in tree hollows and in rotten wood experience very different environmental conditions compared to ground-dwelling beetles. Subcortical spaces and tree hollows can provide more insulation and hence more humid and microclimatically more stable conditions compared to ambient conditions [69,70,71] to which ground dwelling tenebrionids are more directly exposed. At higher elevations, while ground temperatures drop substantially on a daily and seasonal basis, the relatively warmer and more stable microclimate under bark, in tree hollows and in rotten wood could support increased beetle activity and longer growth, thus allowing the presence of relatively large species. While this may be a reasonable explanation for the presence of relatively large species of xylophilous tenebrionids even at higher elevations, it is not sufficient to explain their average increase in body size along the whole gradient. For other insects, it has been postulated that larger body sizes may increase resistance to starvation, as energy stores increase with size faster than metabolic rate [72]. This explanation might be evoked to explain the increasing body size of geophilous species above 1100 m. In the case of xylophilous species, another explanation can also be proposed. Data on phenological patterns in the tenebrionid species of Latium indicate that adult beetles are rarely found during winter among geophilous species, whereas it is easy to find overwintering adults among xylophilous species (Fattorini, pers. observ.). It can be speculated that insulation provided by the microhabitats used by xylophilous species allows them to overwinter more easily compared with geophilous species. The need to store energy for overwintering may determine an increase in average body size with increasing elevation in these tenebrionids. Thus, while, in general, smaller beetles might be favored at higher elevations because of reduced resource availability, the microclimatic conditions and selective pressures within the specific habitats of xylophilous species can override this general trend.

According to Bergmann’s rule hypothesis, larger bodies are favored in colder climates because a lower surface area-to-volume ratio reduces heat dissipation [73,74]. Within a given species, surface area-to-volume ratios are expected to correlate strongly with body length, mass, and volume, as shape usually exhibits only negligible intraspecific variation. However, when species assemblages are considered, the average values of surface area-to-volume ratios are not necessarily correlated with body length, mass, and volume because different species may differ profoundly in their shape. Thus, while measures of body length, mass, and volume may be used as proxies for surface area-to-volume ratios in intraspecific studies, they may be inadequate in interspecific studies. When the surface area-to-volume ratios were used, tenebrionid beetles showed a declining pattern with increasing elevation for both the total species and for the geophilous and xylophilous species separately. These results suggest that increasing elevation favors species with a lower surface area-to-volume ratio (independently from their size) since they loss less heat. Overall, the general reduction in average body size (mass and volume) and surface area-to-volume ratio with increasing elevation supports the hypothesis (H1) that resource availability limits body size and the need for heat conservation is more important than rapid heating and cooling in tenebrionid beetles in general and in geophilous species in particular (up to a certain elevation). In xylophilous species (and in geophilous species above a certain elevation), the general increase in average body size (length, mass and volume) and reduction in surface area-to-volume ratio with increasing elevation support the hypothesis (H3) that resource availability does not limit body size and heat conservation is more important than rapidly heating and cooling. This may be explained by the climatically more stable conditions of the microhabitats occupied by xylophilous insects, which allow them to rely on more constant resources, and for the importance of energy conservation for overwintering, while for high elevation geophilous species relatively larger sizes might be correlated with increased resistance to starvation.

The results obtained in the present study suggest that as elevational patterns in body size are sensitive to species ecology, using species groupings only based on taxonomy (as in the case of the analyses involving all species together) might lead to overlook the importance of species lifestyle in generating Bergmann’s and converse Bergmann’s patterns. By contrast, surface area-to-volume ratios present identical patterns in both geophilous and xylophilous beetles, which makes this measure insensitive to species ecology.

The approach used in this study is simple and easy to reproduce in other insect groups from areas for which distributional data are sufficiently accurate. It would be interesting to extend these results to other insect faunas to investigate the generality of the results obtained in the present study. A useful comparative approach should involve (1) the analysis of different groups in the same area to test the influence of their ecology on the associated patterns of variation in body size and surface area-to-volume ratio and (2) the analysis of the same group in different areas, to test the possible influence of differences in geographical conditions.

An important limit of the present study is that measures of body mass, volume and surfaces were obtained through approximations. Body mass was calculated using allometric equations and not from direct measurements. Thus, the use of direct measures of body mass would be desirable in future research. Surface area and volume values were obtained from three linear measures under the assumption that tenebrionid bodies can be approximated by an ellipsoid. While this appears reasonable, future research might benefit from more precise measures, such as 3d modeling [75]. Finally, it should be noted that the data used in this research refer to interpolated species presences along a regional elevational gradient, which does not mean that species present in a given elevational band really co-occur in the same place. A more accurate analysis might be developed in the future by using data at a community level and local measures of temperatures.

## 5. Conclusions

This study demonstrates that interspecific average values of surface area-to-volume ratio of tenebrionid beetles in central Italy decline with increasing elevation at a regional scale, thus indicating that the need for heat conservation is more important than rapid heating and cooling. This pattern was recognized both in geophilous and xylophilous species. These two groups, however, showed different patterns of elevational variation in body size (mass and volume). Body size declined with increasing elevation in geophilous species (converse Bergmann pattern) up to a certain elevation but increased (Bergmann’s rule) along the entire elevational gradient in xylophilous species (which typically live under bark). As resources are assumed to decline with increasing elevation, these findings suggest that a reduction in resource availability limits body size in geophilous species, while xylophilopus species, which benefit from more constant resources, need more mass to have energy for overwintering. Overall, these results highlight the importance of considering species ecology when investigating Bergmann’s rule in insects.

## Figures and Tables

**Table 1:** Relationship between measures of body size and elevation (in m) for tenebrionid beetles in Latium (Central Italy). Degrees of freedom for *F* were: 1, 22 for all species together and geophilous species separately; 1, 20 for xylophilous species separately; 1, 9 for geophilous species < 1100 m; 1, 11 for geophilous species > 1100 m.

Measure	Group	Intercept	Slope	*R* ^2^	*F*	*p*
Body length (mm)	All	9.578 ± 0.159	0.0008 ± 0.0001	0.689	48.7	5.315 × 10^−7^
	Geophilous	11.108 ± 0.309	−0.0003 ± 0.0002	0.098	2.4	0.136
	Geophilous (<1100 m)	11.399 ± 0.471	−0.0005 ± 0.0007	0.043	0.4	0.541
	Geophilous (>1100 m)	7.972 ± 0.396	0.0013 ± 0.0002	0.769	36.5	8.393 × 10^−5^
	Xylophilous	6.307 ± 0.468	0.0040 ± 0.0004	0.869	132.2	2.875 × 10^−10^
Body mass—fresh (mg)	All	167.988 ± 8.109	−0.038 ± 0.006	0.653	41.4	1.798 × 10^−6^
	Geophilous	210.425 ± 16.665	−0.078 ± 0.012	0.655	41.8	1.666 × 10^−6^
	Geophilous (<1100 m)	247.387 ± 26.337	−0.130 ± 0.042	0.522	9.8	0.012
	Geophilous (>1100 m)	44.723 ± 5.610	0.013 ± 0.003	0.609	17.1	0.002
	Xylophilous	62.444 ± 15.261	0.086 ± 0.012	0.718	50.9	6.497 × 10^−7^
Body mass—dry (mg)	All	47.550 ± 1.820	−0.005 ± 0.001	0.443	17.5	0.0004
	Geophilous	59.470 ± 3.952	−0.016 ± 0.003	0.601	33.1	8.687 × 10^−6^
	Geophilous (<1100 m)	66.658 ± 6.174	−0.025 ± 0.010	0.428	6.7	0.029
	Geophilous (>1100 m)	18.663 ± 2.153	0.006 ± 0.001	0.678	23.1	0.0005
	Xylophilous	18.038 ± 3.997	0.029 ± 0.003	0.810	85.3	1.181 × 10^−8^
Body volume (mm^3^)	All	175.287 ± 9.735	−0.019 ± 0.007	0.247	7.2	0.013
	Geophilous	247.812 ± 18.539	−0.063 ± 0.013	0.505	22.4	0.0001
	Geophilous (<1100 m)	281.646 ± 28.567	−0.105 ± 0.045	0.376	5.4	0.045
	Geophilous (>1100 m)	55.261 ± 12.196	0.041 ± 0.007	0.770	36.9	8.054 × 10^−5^
	Xylophilous	62.561 ± 11.732	0.066 ± 0.009	0.719	51.2	6.275 × 10^−7^

**Table 2:** Relationship between surface area-to-volume ratio (mm^−1^) and elevation (in m) for tenebrionid beetles in Latium (Central Italy). Degrees of freedom for *F* were 1, 22 for all species together and for geophilous species, and 1, 20 for xylophilous species.

Group	Intercept	Slope	*R* ^2^	*F*	*p*
All	2.405 ± 0.046	−5.821 × 10^−4^ ± 3.344 × 10^−5^	0.932	303.1	2.371 × 10^−14^
Geophilous	2.094 ± 0.052	−4.455 × 10^−4^ ± 3.754 × 10^−5^	0.865	140.8	4.933 × 10^−11^
Xylohiplous	2.840 ± 0.091	−8.223 × 10^−4^ ± 7.146 × 10^−5^	0.869	132.4	2.837 × 10^−10^

## Data Availability

Data supporting reported results are included as Supplementary Materials.

## Supplementary Materials

The following supporting information can be downloaded at: https://www.mdpi.com/article/10.3390/insects15090673/s1, Table S1: Values of body length, width and height, volume, surface area, surface area-to-volume ratio, fresh and dry mass for the tenebrionid beetles of Latium, their lifestyle and elevational distribution; Table S2: Average values of body length, fresh and dry mass, volume and surface area-to-volume ratio for the tenebrionid beetles of Latium at different elevations. Figure S1: Overall relationships (regression lines) of average values of body length (a), body mass (fresh) (b), body mass (dry) (c) and body volume (d) with elevation for geophilous tenebrionid beetles in Latium (Central Italy).
