# Supplementary figures and images for: An Interspecific Assessment of Bergmann’s Rule in Tenebrionid Beetles (Coleoptera, Tenebrionidae) along an Elevation Gradient
Source: Insects. 2024 Sep 5;15(9):673. doi: 10.3390/insects15090673 (PMC11432099; doi:10.3390/insects15090673)

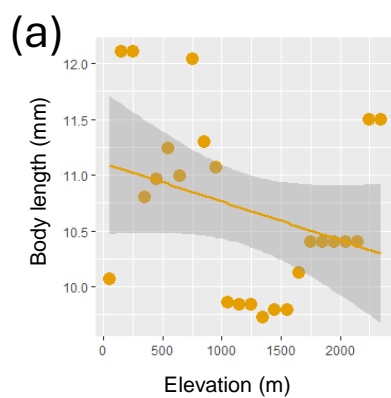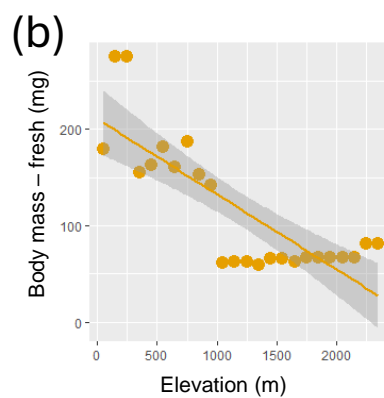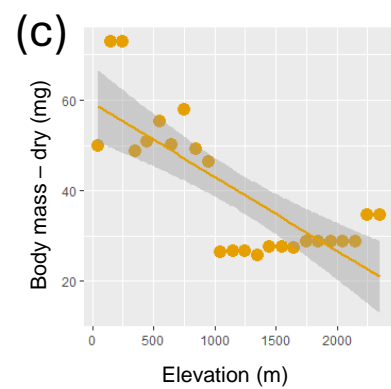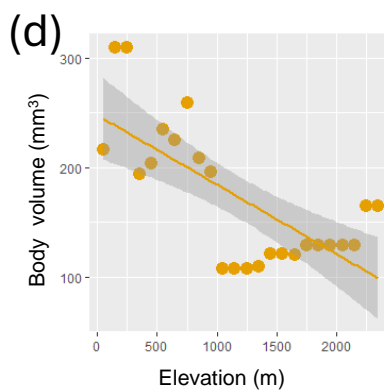

Supplement: Supplementary file 1 [file insects-15-00673-s001.zip › FigS1.pdf]
